# A Reference Database of Standardised Continuous Lumbar Intervertebral Motion Analysis for Conducting Patient-Specific Comparisons

**DOI:** 10.3389/fbioe.2021.745837

**Published:** 2021-09-27

**Authors:** Alexander Breen, Diana De Carvalho, Martha Funabashi, Greg Kawchuk, Isabelle Pagé, Arnold Y. L. Wong, Alan Breen

**Affiliations:** ^1^ AECC University College, Bournemouth, United Kingdom; ^2^ Division of Community Health and Humanities, Faculty of Medicine, Memorial University of Newfoundland, St. John’s, NL, Canada; ^3^ Division of Research and Innovation, Canadian Memorial Chiropractic College, Toronto, ON, Canada; ^4^ Département de chiropratique, Université du Québec à Trois-Rivières, Trois-Rivières, QC, Canada; ^5^ Department of Physical Therapy, Faculty of Rehabilitation Medicine, University of Alberta, Edmonton, AB, Canada; ^6^ Department of Rehabilitation Sciences, The Hong Kong Polytechnic University, Hong Kong, SAR China; ^7^ Faculty of Science and Technology, Bournemouth University, Poole, United Kingdom

**Keywords:** back pain, videofluoroscopy, lumbar spine, intervertebral motion, kinematics, reference database, instability

## Abstract

Lumbar instability has long been thought of as the failure of lumbar vertebrae to maintain their normal patterns of displacement. However, it is unknown what these patterns consist of. Research using quantitative fluoroscopy (QF) has shown that continuous lumbar intervertebral patterns of rotational displacement can be reliably measured during standing flexion and return motion using standardised protocols and can be used to assess patients with suspected lumbar spine motion disorders. However, normative values are needed to make individualised comparisons. One hundred and thirty-one healthy asymptomatic participants were recruited and performed guided flexion and return motion by following the rotating arm of an upright motion frame. Fluoroscopic image acquisition at 15fps was performed and individual intervertebral levels from L2-3 to L5-S1 were tracked and analysed during separate outward flexion and return phases. Results were presented as proportional intervertebral motion representing these phases using continuous means and 95%CIs, followed by verification of the differences between levels using Statistical Parametric Mapping (SPM). A secondary analysis of 8 control participants matched to 8 patients with chronic, non-specific low back pain (CNSLBP) was performed for comparison. One hundred and twenty-seven asymptomatic participants’ data were analysed. Their ages ranged from 18 to 70 years (mean 38.6) with mean body mass index 23.8 kg/m^2^ 48.8% were female. Both the flexion and return phases for each level evidenced continuous change in mean proportional motion share, with narrow confidence intervals, highly significant differences and discrete motion paths between levels as confirmed by SPM. Patients in the secondary analysis evidenced significantly less L5-S1 motion than controls (*p* < 0.05). A reference database of spinal displacement patterns during lumbar (L2-S1) intersegmental flexion and return motion using a standardised motion protocol using fluoroscopy is presented. Spinal displacement patterns in asymptomatic individuals were found to be distinctive and consistent for each intervertebral level, and to continuously change during bending and return. This database may be used to allow continuous intervertebral kinematics to drive dynamic models of joint and muscular forces as well as reference values against which to make patient-specific comparisons in suspected cases of lumbar spine motion disorders.

## Introduction

Pathological spinal motion, or lumbar instability, has long been thought of as the failure of the lumbar spine to maintain its normal pattern of displacement (Panjabi, 1992). However, it is currently unclear what this normal pattern actually consists of, as the motion segments of the spine are sited deep within the body, making them practically impervious to objective biomechanical measurement in living people. This tends to deny clinicians the tools to investigate relationships between symptoms and intrinsic biomechanics and constrains the identification of biomechanical markers for spinal pain. Given that the spine has a complex dynamic role in the normal activities of daily living, recent proposals for future directions in spine biomechanics research have included the recommendation that *“The dynamic properties of the* (functional spinal unit) *FSU … should be a focus of future research efforts as they are likely very relevant to the in vivo situation.”* (Oxland, 2016). As non-invasive, *in vivo* measurement of the dynamic properties of the FSU generally requires imaging, precision imaging measurement of *in vivo* segmental spine dynamics is critical for gaining an understanding of spine biomechanics that could be applied in patient-specific assessments.

Spine biomechanics also increasingly involves biomechanical modelling, where *“the importance of verification, validation and sensitivity testing in computational studies within the field of biomechanical engineering”* has been highlighted (Jones and Wilcox, 2008). These models are sometimes utilized to estimate muscle and inter-joint forces within the lumbar spine, as they provide a relatively inexpensive and efficient method to estimate specific characteristics that are not otherwise possible or practical to measure *in-vivo*. However, while there are studies that provide *in vivo* information about intradiscal pressures, forces, and moments transmitted *via* instrumented vertebral implants, there is a lack of reference information with respect to multilevel continuous intervertebral motion for use in dynamically modelling loads (Dreischarf et al., 2016).

Although thorax and pelvis kinematics, used to drive such models, have often been measured using skin-based motion capture, the inherent errors associated with the proper identification of underlying bony landmarks mean that skin-based tracking is rarely used for measuring the motion of individual vertebrae (Eskandari et al., 2017). Instead, the kinematics of the lumbar vertebrae are often approximated from their segmental contributions to flexion motion based on static end-of-range radiographs. These contributions are then applied to the measured kinematics of the thorax-pelvis to estimate joint motion in the lumbar spine. However, it has been questioned as to whether this accurately represents vertebral orientation, for example, during dynamic bending tasks (Nagel et al., 2014; Aiyangar et al., 2015).

In addition, “Despite RoM being a simple metric that could be easily estimated within a clinical setting, it does not convey the contribution over time of the related segments/joints to the movement performed, compensatory actions nor the movement variability, thus limiting our understanding of movement strategies” (Papi et al., 2018). However, lumbar segmental contributions to motion, sometimes referred to as “spinal rhythms”, have been demonstrated to change during simple tasks such as controlled flexion and return motion, (Aiyangar et al., 2015; Breen and Breen, 2020), and even during passive movement, where there is no measurable muscle activation (Breen and Breen, 2018). As such, physical and computational models that are validated using only end range of motion data may not accurately reproduce dynamic *in vivo* motion. Indeed, this may be one of the major causes of the large differences found in inter-joint and muscle forces when comparing models driven by generic patterns of rotational displacement in the lumbar spine and those based on kinematics acquired from dynamic imaging techniques (Eskandari et al., 2017; Byrne et al., 2020).

With advancements in imaging and object tracking technologies, continuous assessment of intersegmental spine motion during bending using quantitative fluoroscopy (QF) has been demonstrated to be relatively accurate and repeatable (Breen et al., 2019b). Thus, using QF for inter-image vertebral body tracking to quantify spine motion has allowed continuous intervertebral lumbar motion measurement *in-vivo*. However, the precision (and therefore the application) of dynamic models that integrate anthropometric and kinematic data will be limited if there is uncontrolled variation in subjects’ motion behaviour (Magee, 2015).

In previous work using QF, where both the motion task and the analysis were highly standardised for range and velocity, some intervertebral motion sharing characteristics in the lumbar spine were found to be significantly different in chronic, nonspecific back pain (CNSLBP) patients compared with asymptomatic controls, indicating their eligibility to be considered as pain biomarkers (Breen and Breen, 2018; Breen and Breen, 2020). As some of these measurements were found to be relatively stable over 6 weeks in an asymptomatic population, this made these measures potentially suitable for use in outcome and prognostic studies. This, however, highlights the need for a reference database of normal values against which individuals could be compared (Breen et al., 2019a; Breen et al., 2019b). As the differences between patients and controls found in these studies were in terms of continuous proportional motion sharing parameters, it was decided to formulate a normative Reference Database of these as information against which patient-specific comparisons could be made.

The present study therefore aimed to create a normative set of values for flexion and return dynamic lumbar segmental rotational contributions from a sizeable population base that could be used to drive future models. To support future patient-specific comparative studies and inform such musculoskeletal models, the project aimed to employ a standardised protocol, rather than a free-bending one, and to identify the intersegmental contributions to motion from L2-S1 during weight bearing flexion and return in asymptomatic individuals.

Given that more recent studies have focused on the return paths of lumbar flexion separately, to support dynamic loading models during lifting (Aiyangar et al., 2015; Pavlova et al., 2018), the motion was separated into the flexion phase, and the return to neutral phase for analysis. In addition, as proportional motion has been found to discriminate patients and controls in the past (Breen and Breen, 2018; Breen et al., 2018; Breen and Breen, 2020) but has not yet been analysed across the time series, this analysis protocol was also applied in a further secondary analysis of a matched Patient-control subgroup.

## Materials and Methods

The methods used for image acquisition and analysis in this project were agreed by an international forum of QF users in 2009 (Breen et al., 2012), and applied in the present Reference Database study. The participants in the Forum were the only four groups of QF users known to the authors in 2009, who all employed automated image registration and/or tracking for extracting vertebral kinematics data and used well documented data collection protocols. The focus of the Forum was to agree a standard protocol for data collection and analysis that could be employed efficiently for investigating and comparing symptomatic and asymptomatic participants for clinical investigations and research.

### Participants

A convenience sample of 131 asymptomatic volunteer participants was recruited to the Reference Database study from staff, students and visitors at the AECC University College (Bournemouth, United Kingdom) between July 2011 and July 2020. Participants were included based on the following inclusion criteria: between 21 and 80 years old, self-reported body mass index less than 30 kg/m^2^ (to ensure image quality), free of pain on the day of testing, free of any back pain that limited normal activity for more than 1 day in the previous year, no history of abdominal surgery or spondylolisthesis, no medical radiation exposure of >8 mSv in the previous 2 years (self-identified by pre-study questionnaire detailing recent medical imaging), and not currently pregnant. Ethical approval was obtained from the United Kingdom National Research Ethics Service (SouthWest 3, 10/H0106/65) and written Informed consent was obtained from all participants prior to inclusion in the study.

For the Patient-control subgroup study, 8 patients without any obvious mechanical disruption (for example surgery or spondylolisthesis), who had been referred for QF imaging to investigate CNSLBP using the same imaging protocol, were recruited. Their imaging results were compared to those of 8 of the asymptomatic controls, following written informed consent to inclusion on the study. The controls were chosen from the Reference Database as being of similar age, sex and BMI to the patients. Their demographic information is shown in Table 1.

**TABLE 1:** Participant characteristics (mean and SD).

**Reference database**		**Subgroup study**		
		**Controls**	**Patients**	**2-tailed p**
N	127	8	8	
Females (%)	62 (48.8)	3 (38.8)	3 (38.8)	0.99
Age (years)	38.6 (13.8)	48.1 (13.4)	48.8 (14.4)	0.93
Height (m)	1.73 (0.09)	1.70 (0.1)	1.70 (0.1)	0.71
Weight (kg)	71.6 (12.7)	74.5 (12.7	75.4 (10.5)	0.41
BMI	23.8 (2.9)	25.8 (6.5)	25.3 (5.3)	0.42

SD: standard deviation; m: meters; kg: kilograms; BMI: body mass index.

### Reference Database Study Sample Size

The design criterion for determining the sample size needed to establish a credible 95% reference interval (RI) is the ratio of the confidence interval (CI) width on the RI cut-point to the RI width. Practical values for this ratio suggested by Linnet range from 0.1 to 0.3 (Linnet, 1987). Using a conservative ratio of 0.15, with a 90% cut-off CI and a single 95% upper RI cut-point, we required 134 participants (SSS software v.1, Wiley-Blackwell, Chichester United Kingdom). To allow for tracking failure in approximately 10% of sequences, we rounded the sample size up to 148. However, assuming a non-Gaussian distribution for at least some of the reference data, we employed the non-parametric RI methodology recommended in the Clinical Laboratory Standards Institute guidelines, for which the minimum recommended sample size is 120 (CLSI, 2008). This was therefore selected as the minimum population for the Reference Database study.

### Data Collection

**FIGURE 1:**
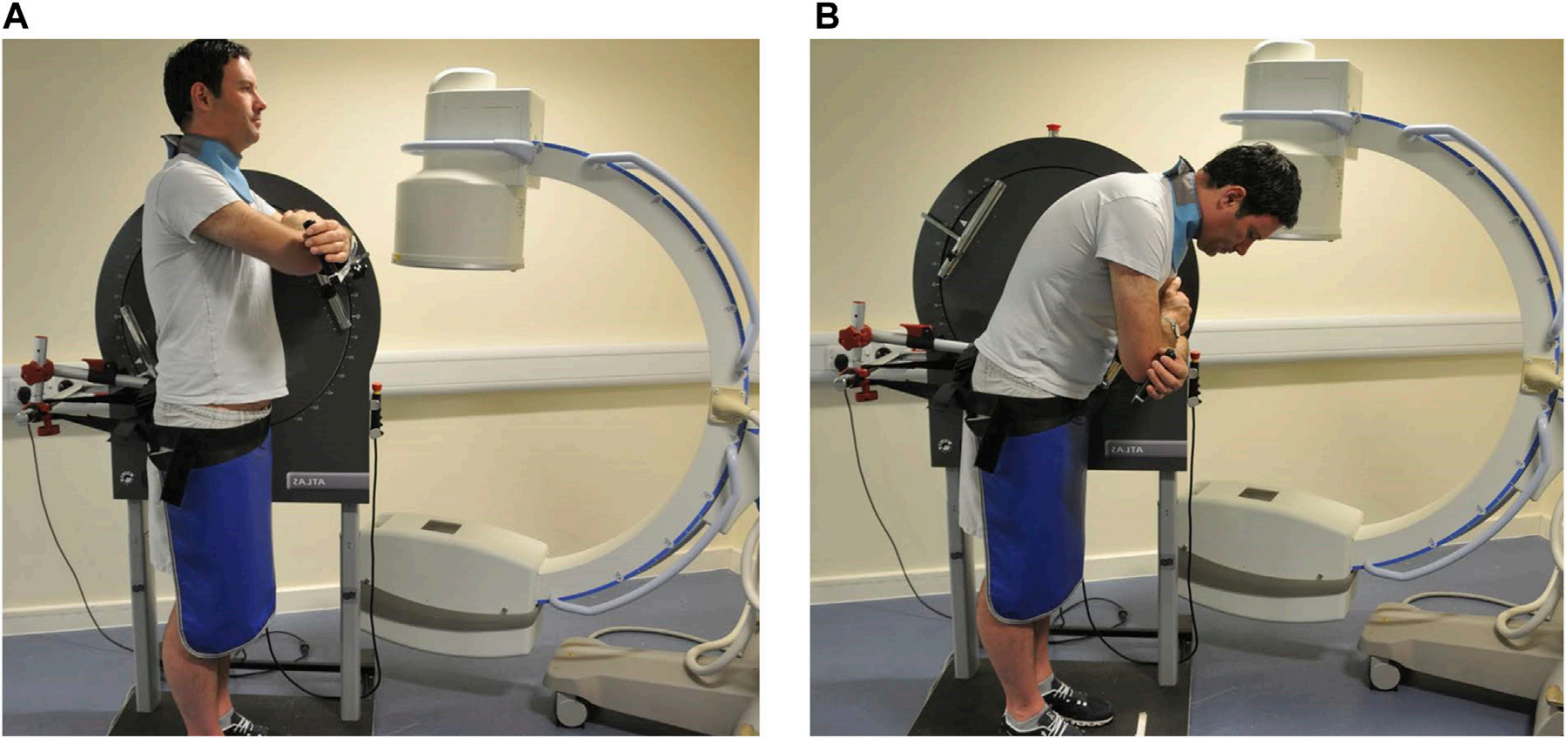
Motion protocol used for fluoroscopic image acquisition (Courtesy Atlas Clinical Ltd., Lichfield, United Kingdom) **(A)** upright **(B)** flexed.

The QF protocol for image acquisition and analysis procedures as been detailed in previous studies (Breen et al., 2012; Breen and Breen., 2018; du Rose et al., 2018; Breen and Breen, 2020). In brief however, participants were guided through a standard active weight-bearing flexion and return motion task. This was designed to reduce behavioural variations in participant bending, while controlling the speed and range of motion in a reproducible way. During this controlled motion, low dose fluoroscopic recordings of L2-S1 levels during continuous motion were acquired using a Siemens Arcadis Avantic digital C-arm fluoroscope (Siemens GMBH) at 15 frames per second. To achieve this, participants stood with their right-hand side next to a motion testing platform (Atlas Clinical Ltd. Lichfield, United Kingdom), which guided them through a 60° bending arc at 6°/s during both flexion and return phases (Figure 1). Participants were positioned in a comfortable upright stance with the centre of rotation of the motion platform in line with the disc space between the third and fourth lumbar vertebrae (This position was confirmed by single short pulse fluoroscopic images and the use of radiopaque markers temporarily aligned with the platform’s centre of rotation.) A sacral brace and a belt around the hips of participants were used to minimise pelvic motion and keep the spine in the field of view throughout the bending sequence. This was to ensure the best field of view for all the segments to be conveniently imaged throughout the whole range of motion.

Before the acquisition of the QF images, participants undertook 3 practice bends. These standing movements, bending to 20° flexion and return, were followed by 40-degree and 60-degree bends. This ensured that participants could perform their recorded motion confidently and smoothly.

### Intervertebral Motion Analysis

A previously validated semi-automated tracking process was used to determine the position of each vertebra (L2, L3, L4, L5 and S1) within each image recorded during the flexion and return trials (Breen et al., 2012). This process has been shown to have an accuracy for measuring intervertebral RoM of 0.52°, (du Rose and Breen, 2016), inter-and intra-observer repeatability ranging from ICC 0.94–0.96 and SEM 0.23°–0.61° and acceptable intra-subject repeatability (ICC 0.96, MDC over 6 weeks, 60%) (Breen et al., 2006; du Rose and Breen, 2016; Breen et al., 2019b).

**FIGURE 2:**
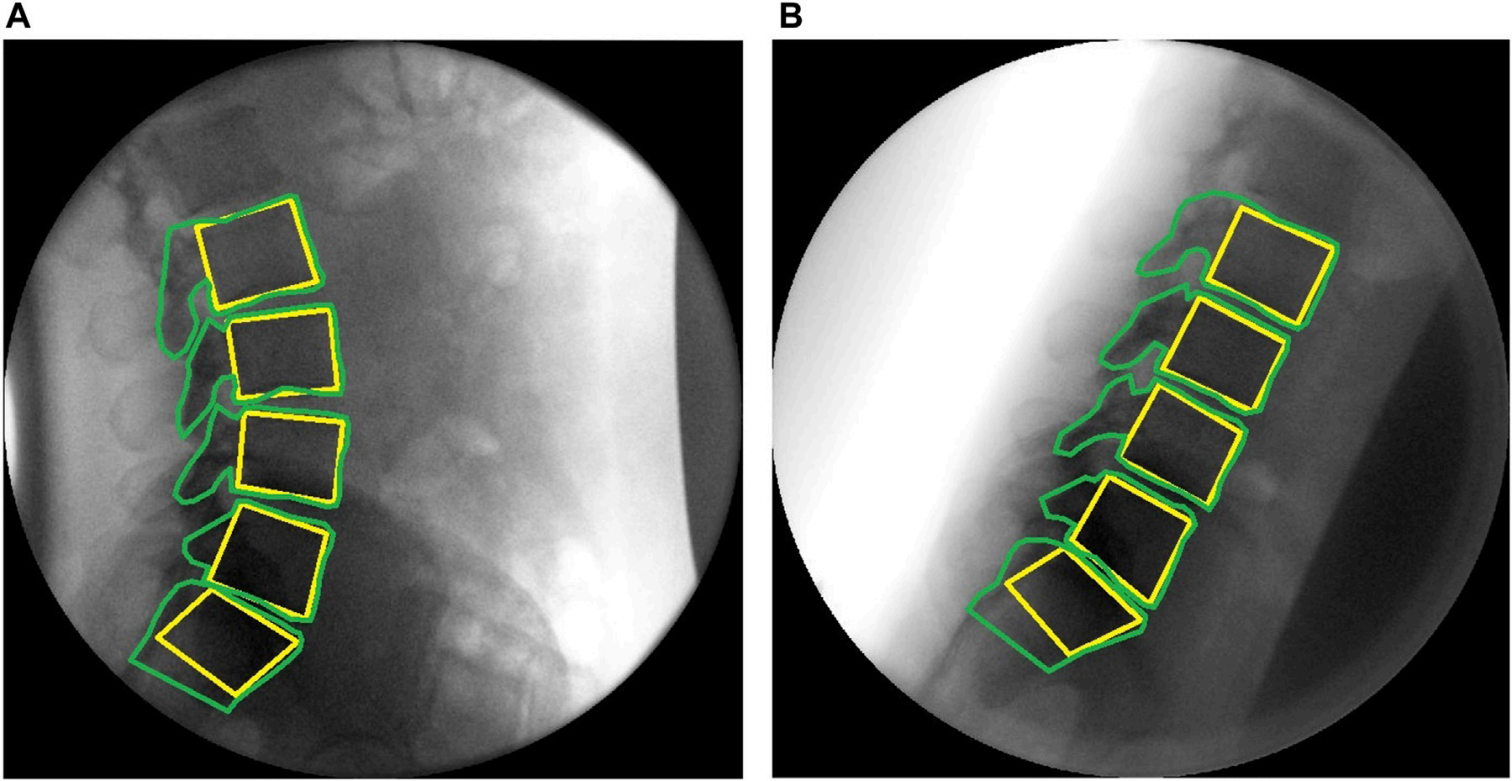
Sagittal plane fluoroscopic images of the lumbar spine with computer templates **(A)** upright **(B)** flexed.

Rotations were extracted from the positions for each of the tracked vertebrae (L2, L3, L4, L5 and S1, Figure 2) in each of the QF images throughout the flexion and return movement. Changes in the intervertebral angle from the starting position at each level (L2-L3, L3-L4, L4-L5, and L5-S1) over time were then computed. The motion outputs were separated into two phases, the flexion phase, and the return to neutral phase. To standardise the representation of motion across all participants, the L2-S1 angle was normalized to a percentage of its range of motion (RoM). Thus, during the flexion phase, standing was defined as 0% RoM and maximum flexion as 100% RoM, while in the return phase, 0% RoM was defined as maximum flexion and 100% RoM as being returned to the original reference position.

Changes in intervertebral angles were then interpolated to obtain each intervertebral motion segment’s rotation for every 1% increment of the L2–S1 RoM. The segmental contribution of each intervertebral level as a percentage of the change in L2–S1 angle was then computed at every increment.

### Statistical Analysis

For the Reference Database study, the share of intervertebral segmental motion was calculated for all participants for each level throughout the bending task. Statistical Parametric Mapping (SPM) was then used to compare the whole kinematic time-series between levels’ contributions to motion for both the flexion and return sequences (Friston et al., 2007). SPM analysis is an open-source spm1d package (available from www.spm1d.org) based on Random Field Theory, and has been validated for 1D data (Adler et al., 2007; Pataky et al., 2016; Pataky, 2016). Following normality testing, custom Python programs (Python version 3.8) were used to conduct parametric two-tailed, two-sample t-tests across the time series. Statistical significance occurs when the SPM curves cross the critical threshold node at any time, taking into account that each time point is related to those on either side (Friston et al., 2007; Papi et al., 2020). Where multiple adjacent points of the SPM curves exceeded the critical threshold, the associated *p*-values were calculated using Random Field Theory.

For the Patient-control secondary analysis, SPM analysis was conducted using non-parametric two tailed t-tests. This compared segmental contributions to bending between patients and controls throughout the motion. Previous measures of segmental contribution have been shown to have high observer reliability and acceptable intrasubject repeatability over 6 weeks (Breen et al., 2019b; To et al., 2020).

## Results

For the Reference study, 131 participants were imaged. Four participants were excluded due to tracking errors of at least 1 vertebra. Full data sets were therefore obtained from 127 participants. Tables containing the Reference Database, detailing the mean and 95%CI for the continuous proportional segmental motion for flexion and return motion plus the Patient-Control secondary analysis data can be found in Supplementary Material I.

Reference Database study participants received a mean (upper third quartile) effective dose of 0.27 mSv (0.31) while secondary analysis patients received 0.26 mSv (0.30) for this investigation. These values are approximately one quarter of the dose of a conventional plain radiographic examination of the lumbar spine (Mellor et al., 2014).

For the Patient-control sub-study, 8 chronic back pain patients and 8 controls were imaged (43.8% female, mean age 48.1 (controls) and 48.8 years (patients). Thus, the sub-study participants were approximately 10 years older than those in the Reference Database study who had a mean age of 38.6. This was the only substantial difference between the studies.

### Kinematics

The maximum intervertebral ranges throughout flexion and return motion (means) for the Reference Database study group and the Patient-control sub-study group are shown in Table 2. Maximum change in L5-S1 RoM was significantly less than the controls in the Patient-control sub-study.

**TABLE 2:** Mean maximum intervertebral rotational ranges (mean and SD).

**Reference database**		**Subgroup study**		
		**Controls**	**Patients**	**2-tailed p**
RoM L2-3	9.5 (3.87)	10.2 (1.4)	8.9 (5.2)	0.46
RoM L3-4	10.6 (2.96)	11.5 (2.8)	10.1 (2.8)	0.21
RoM L4-5	10.4 (3.93)	11.9 (3.5)	8.7 (1.1)	0.21
RoM L5-S1	5.7 (5.60)	7.2 (3.9)	3.2 (2.9)	0.05
RoM: range of motion (degrees)				

**FIGURE 3:**
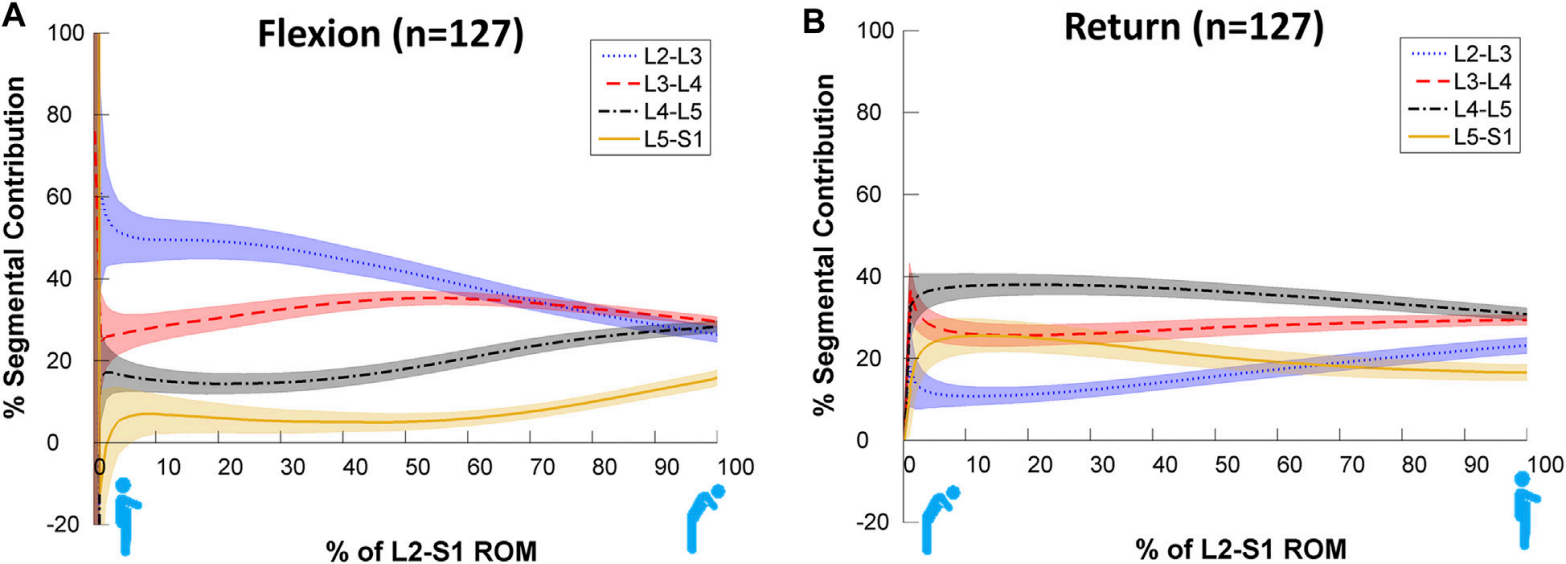
Average segmental contribution to lumbar flexion [**(A)**: Flexion and **(B)**: return to standing] with 95% confidence intervals (shaded areas) in the Reference Database cohort.

Figure 3 shows statistically significant differences in contributions to bending during the motion, both between and within levels. Each intervertebral level had its own characteristic motion signature across the Reference Database study population, with significant differences (*p* < 0.05, noted from the lack of overlap of the 95%CI bands) between each level’s contribution throughout most of the motion. It is also notable that these paths are in a state of constant change as the motion progresses, although all levels exhibit more uniform motion sharing in the return phase than in the flexion phase. In addition, there is a negative contribution to motion of L5-S1 at the beginning of flexion (Figure 3A). This is expected as participants attempt to move their hips back to keep the centre of mass over the feet.

**FIGURE 4:**
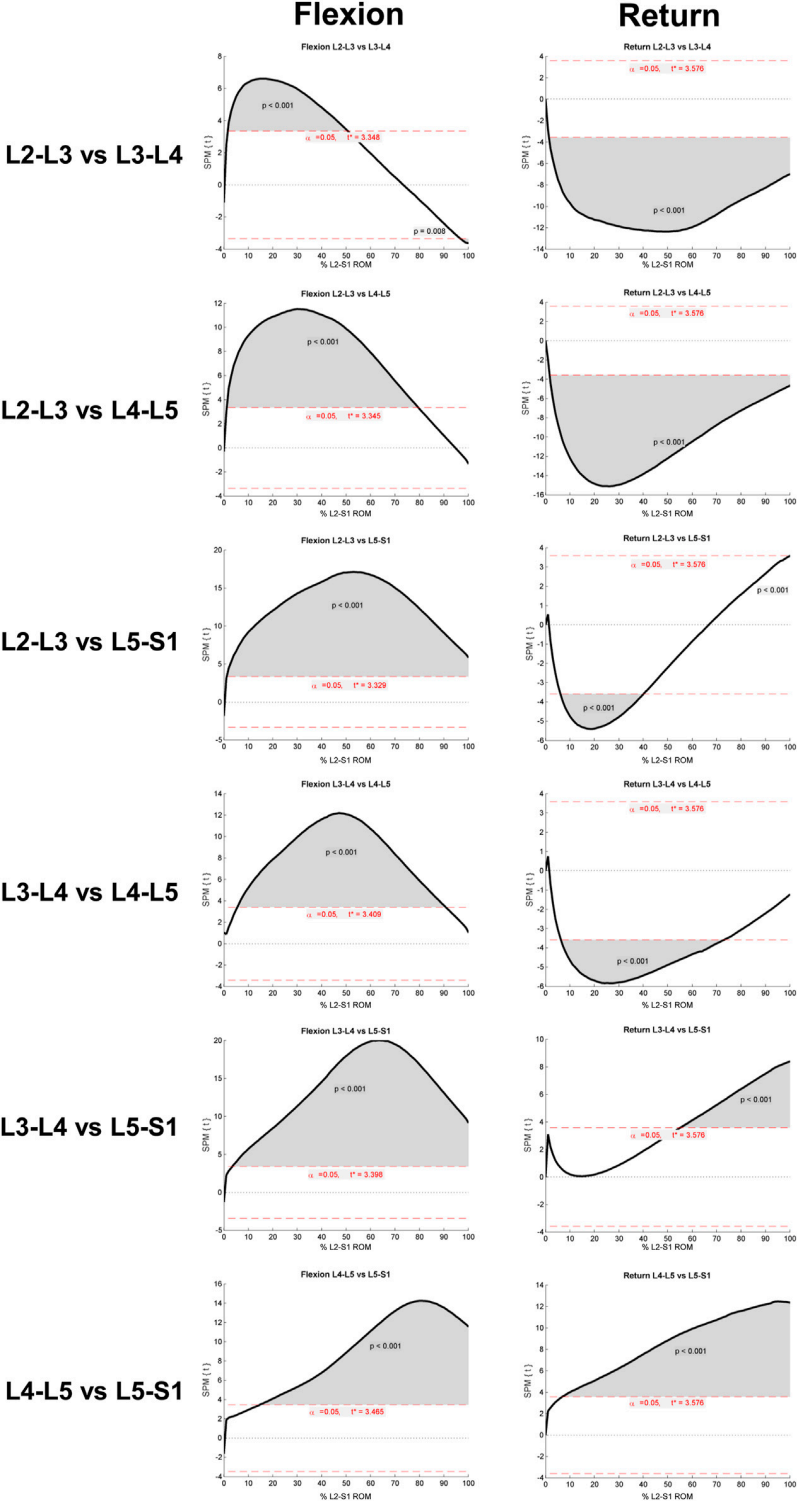
Results of SPM parametric paired t-test (SPM{t}). Each row refers to a different intervertebral joint combination. Supra-threshold clusters indicate significance differences between joint contributions to motion and are shown in grey. The critical threshold is shown as a red dashed line. *Versions of these figures alongside the mean and 95%CI bands can be found in* Supplementary Material II.

The SPM analysis reported in Figure 4 reveals these differences to be highly significant (*p* < 0.001) between levels for almost all data points across the motion for both flexion and return in the Reference Database study cohort, confirming the presence of discrete motion paths for each motion segment. During the outward flexion phase of movement, the superior lumbar motion segment of each pair (Figure 4) consistently contributed more to the range of motion, exceeding the critical value for 50–99% of the task. In addition, the L2-3 vs. L3-4 motion segment combination also had a supra-threshold cluster at the end of flexion where the *inferior* motion segment contributed more (*p* = 0.008).

During the return to upright position phase of the task, in 3 of the 6 inter joint combinations, the inferior motion segment of the pair constantly contributed significantly more to the return phase of bending (*p* < 0.001). The exceptions were “L3-4 vs. L5-S1” and “L4-5 vs. L5-S1”, where the superior motion segment contributed a greater amount (*p* < 0001), and at “L2-3 vs. L5-S1”, where initially (between 5–40% of the RoM) L5-S1 contributed more (*p* < 0.001). In the late stages of bending (at approximately 100% of RoM), L2-3 contributed more (*p* < 0.001).

### Patient-Control Secondary Analysis

**FIGURE 5:**
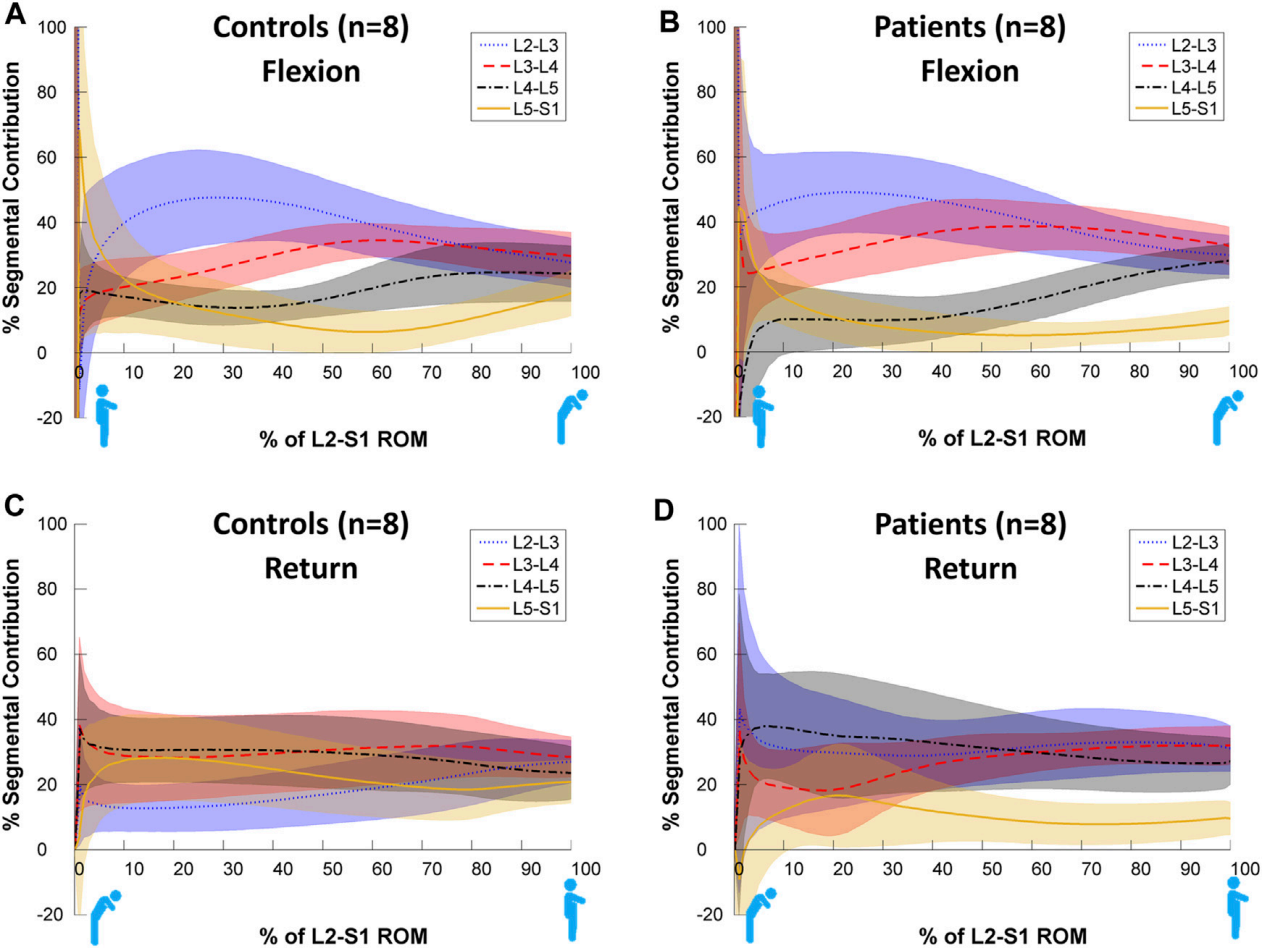
Average segmental contribution to return from flexion with 95% confidence intervals (shaded areas) in the Patient-control sub-study cohorts. **(A)**: Control Flexion, **(B)**: Patient Flexion, **(C)**: Control Return, **(D)**: Patient Return.

The motion contributions in the secondary analysis are shown in Figure 5. These subjectively demonstrate differences in the motion sharing patterns between patients and controls, especially at L5-S1. Verification of these differences can be seen in the non-parametric SPM analysis provided in Supplementary Material III.

**FIGURE 6:**
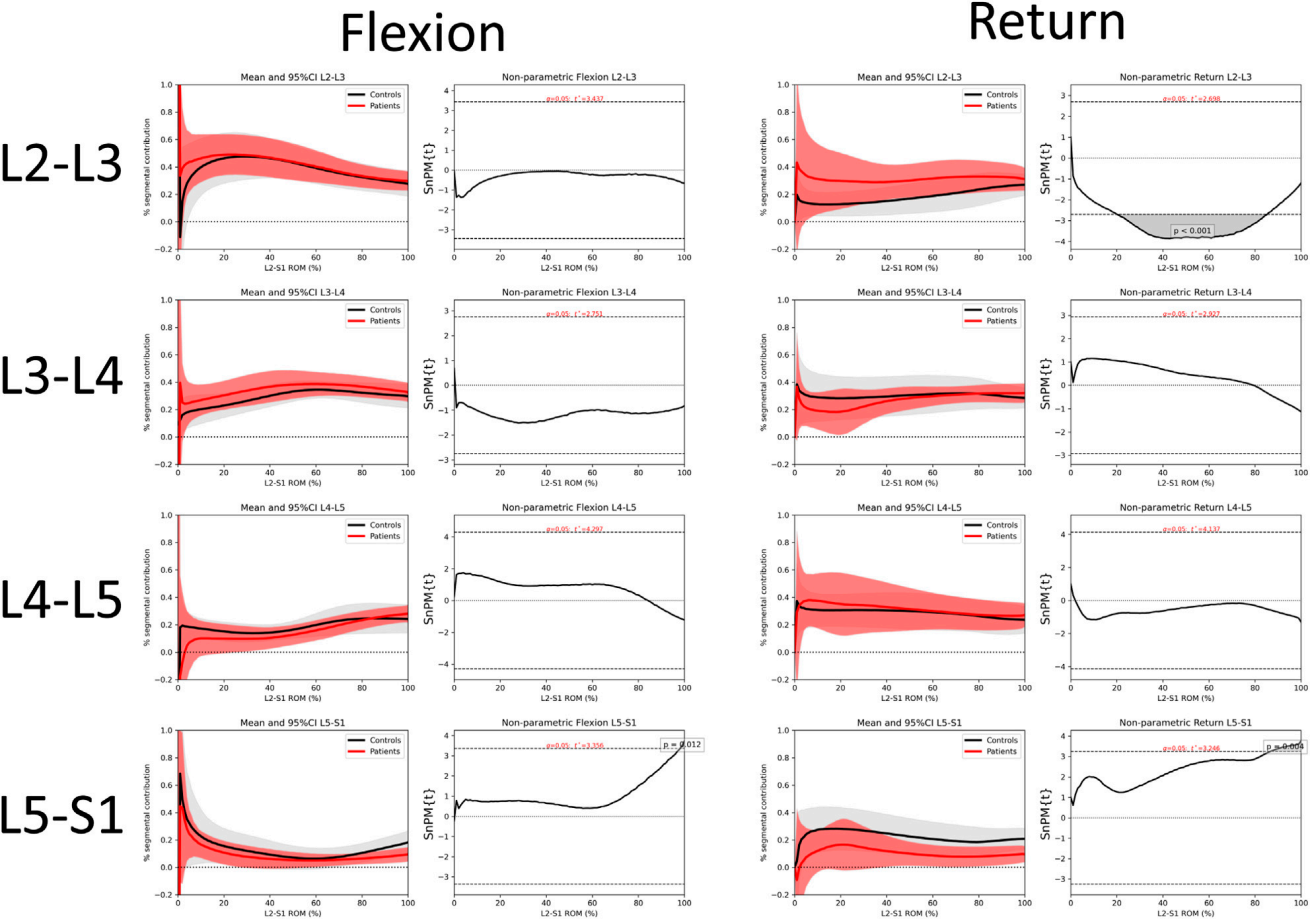
Comparison of intervertebral motion sharing patterns between patients and controls at individual levels for flexion and return with 95% confidence intervals and SPM. Comparisons for all inter-joint combinations are shown in Supplementary Material III.

Figure 6 compares the motion sharing patterns for all 8 patients and 8 controls in the secondary analysis. There was little difference between patients and controls in terms of motion sharing at most intervertebral levels, although these have been found to differentiate patients from controls in passive recumbent studies (Breen and Breen, 2018). However, SPM analyses reveals that there are statistically significant differences between the groups’ motion share at L2-L3 during the return to neutral phase of the task (*p* < 0.001) and at the end range of L5-S1 motion (Flexion *p* = 0.012 and Return *p* = 0.004) (Figure 6).

## Discussion

### Reference Study

To the best of the authors’ knowledge this study reports the largest database of continuous intersegmental lumbar spine kinematics during weight bearing *in-vivo* flexion and return, providing normative reference values for making patient-specific kinematic comparisons, for informing dynamic FE loading models, and to help identify biomarkers for CNSLBP (Zanjani-Pour et al., 2018; Breen and Breen, 2020). The Reference Database study used an established standardised protocol to measure the intersegmental contributions to motion from L2-S1 during weight bearing flexion and return bending–unlike most conventional recording of lumbar flexion, which depends on participant co-ordination for its consistency. Using this protocol, continuous change in mean proportional motion share was observed during both the flexion and return phases and revealed significant differences in the motion paths between levels. In addition, while the similarity between this study’s measures of lumbar segmental contributions and previous measures of lumbo-pelvic rhythms are interesting, these are not the same and should not be confused.

It would be appropriate to compare this database with previous fluoroscopy studies, however no one study has applied all the criteria required. We could find only four that attempted to employ completely continuous motion analysis (Okawa et al., 1998; Harada et al., 2000; Nagel et al., 2014; Aiyangar et al., 2015). This may, in part, account for the failure of studies that reported only quasi-static intersegmental motion to detect variations in the contributions of individual segments during bending (Wong et al., 2006). Only three used proportional motion (Teyhen et al., 2007; Nagel et al., 2014; Aiyangar et al., 2015), and none applied the degree of standardisation of participant motion during imaging used in the present study (Breen et al., 2012). Return phase motion (which is not represented as flexion in reverse) was reported in only 4 (Okawa et al., 1998; Harada et al., 2000; Teyhen et al., 2007; Aiyangar et al., 2015), while only 5 measured all levels from L2-S1 (Takayanagi et al., 2001; Lee et al., 2002; Wong et al., 2006; Ahmadi et al., 2009; Aiyangar et al., 2015). However, when comparing the segmental contributions derived from moderate or maximal flexion studies with continuous intervertebral motion studies, the distribution of sharing was found to be similar (Breen and Breen, 2020). Thus, the quasi-static spine kinematics literature, as reviewed by Widmer et al. (2019) exhibits a degree of consistency with more recent continuous motion studies in terms of lumbar intervertebral motion sharing.

The above considerations, plus the large number of participants in the Reference Database study, may account for the remarkably consistent motion sharing patterns during both outward and return continuous motion, despite some heterogeneity in participant characteristics. Although the age range in our sample was wide (21–70 years), body weight had an upper reference range of only 96 Kg, while weights of up to 119 Kg have been shown to be associated with substantially increased L5-S1 compression in flexed postures (Hajihosseinali et al., 2015). This may affect the segmental contribution at that level and was also noted in relation to RoM in the Widmer et al. (2019) review and in modelling studies by Zander et al. (2002). However, segmental contributions, once thought to be RoM-dependent, did not exhibit this in our Reference Database study, nor in other studies that included all segments from L2 to S1 (Miyasaka et al., 2000; Ahmadi et al., 2009; Aiyangar et al., 2015). Contribution patterns were also distinctly different in flexion and return, as one would expect with different phasing of trunk muscle activation (Ouaaid et al., 2013).

### Patient-Control Sub-Study

The differences between patient and control subgroups in the return phase also seem to complement those previously found in weight bearing studies that combined outward and return motion (Breen and Breen, 2020). Moreover, the standardised image acquisition protocol would seem to make it unlikely that abnormal patterns are attributable to artefact rather than motion pathology. However, it also raises the possibility that other individual factors, such as lumbar geometry, may have an influence, making clinical assessments based on motion sharing patterns alone inadvisable.

The differences between patients and controls in the secondary analysis may reflect differences in the respective roles of the deep multifidus and erector spinae muscle groups in people with CNSLBP (Wallwork et al., 2009) and/or passive tissue restraint. Two trends are particularly apparent in this study of weight bearing motion. Firstly, L5-S1 shares less motion in patients, albeit non-significantly until the end of range. This is also reflected in the reduced RoM of L5-S1. Secondly, L2-3 shares significantly more motion in patients during the return phase, although this is only apparent in the mid-ranges and would not be measurable when merely investigating segmental range. Alterations in the readiness of lumbar joints to move in CNSLBP patients is also reflected clinically in the Kinesiopathological Model of low back pain, and is considered to be an important factor in rehabilitation (Van Dillen et al., 2013).

### Strengths and Limitations

This is the largest dataset available to date to present normative values for continuous segmental contributions to motion in the lumbar spine using variables that have been shown to distinguish patients with CNSLBP from asymptomatic controls (Ahmadi et al., 2009; Breen and Breen, 2020). Moreover, the Patient-control sub-study provides further evidence of a kinematic biomarker for nonspecific back pain. However, standardising the motion protocol involves a trade-off between natural motion and the repeatability necessary to make patient-specific comparisons. In terms of the latter, the methodology used has undergone extensive validation in terms of precision and validity (Breen et al., 2006; Breen and Breen, 2016; Breen et al., 2019b) and has previously been used in preliminary dynamic loading studies using FE modelling (Zanjani-Pour et al., 2018). Thus, further subject-specific estimates of joint loading using dynamic imaging may be expected to improve the sensitivity of subject-specific model-based lumbar spinal loading estimates (Byrne et al., 2020). However, like many other biomechanical studies that compare patients to controls, the sample size of our sub-study was small, which is a limitation that may be mitigated by further replication. In addition, while evidence suggests that magnitude of loading (beyond body weight), *in vivo*, does not have any significant effect on individual segmental contribution to motion (Aiyangar et al., 2015), biomechanical modelling should exercise caution if using this database to model unloaded or excessive loading states.

It would also have been useful for future biomechanical modelling studies if it had been possible to include the whole lumbar spine, but this could not be done owing to the limited image intensifier diameter. This is a problem with most current intensifiers which will be overcome as flat panel machines become more plentiful.

### Future Studies

Further studies are needed, not only to replicate the present study’s findings, but also to explore the effects of other variables, as well as coronal plane motion and passive recumbent motion, where body mass and muscular contractions are mitigated. However, there is still considerable scope for elaboration of motion sharing studies of weight bearing flexion and return. For example, variations in pelvic tilt may be an important source of heterogeneity in light of the variations in the motion segment flexibility that the QF procedure aims to measure (Retailleau and Colloud, 2020).

The specialised motion frame apparatus used in the current work, in addition to standardising the velocity and range of bending, also partially stabilises the sacrum. This increases to varying degrees, the contribution of the lumbar spine to the flexion motion, regardless of the degree of lordosis or sacral inclination. Although the degree of this restraint is not standardised and depends on the individual’s natural lumbo-pelvic contribution to bending, this does not seem to disrupt the consistency of the resulting motion sharing patterns. Nevertheless, there is likely to be some relationship between lumbar sagittal shape and the motion contribution, albeit within the boundaries of the normative ranges of variation. This should be explored. Given that spine shape has been shown to influence a person’s preference for squatting or stooping during lifting tasks, it would be useful to determine the relationships between spine shape and dynamic loading stresses at individual levels based on their contributions to flexion and return motion (Pavlova et al., 2018).

It would also be useful to explore other kinematic indices in terms of motion contributions, as the present database provides only rotational data, and there is evidence that the translational component, although small, also affects inter-segment rotational stiffness (Affolter et al., 2020). However, in a previous study, we did not find it to differentiate nonspecific back pain patients from controls (Breen et al., 2018).

Finally, it is now timely to explore possible relationships between the motion variants that seem to be associated with CNSLBP and possible sources of nociception. As these may not necessarily involve disco-ligamentous micro-strain, it may be useful to explore muscular metabolic pain as a mechanism by including blood flow studies with those of motion contributions during bending.

## Conclusion

In asymptomatic people, provided a standardised QF imaging protocol for measuring continuous proportional lumbar intervertebral motion is used, consistent intervertebral motion patterns are revealed where each level follows its own discrete, level-specific path that changes significantly during the motion. This is proposed to represent the human normative phenotype when using the present imaging protocol. These paths constantly and consistently change as the bending motion progresses, although levels exhibit more uniform motion sharing in the return phase than in the flexion phase. Patients with CNSLBP showed a significantly greater contribution at L2-3 and a significantly smaller contribution at L5-S1 during the return phase.

## Data Availability

The original contributions presented in the study are included in the article/Supplementary Material. Further inquiries can be directed to the corresponding author.

## Supplementary Material

Supplementary Material I Proportional intervertebral motion of individual level L2-S1 motion during forward bending and return in the Reference Database and Patient-control studies.

Supplementary Material II SPM of inter-joint contributions to motion from the Reference Database, together with mean and 95% confidence intervals for each combination.

Supplementary Material III Differences in the motion sharing patterns for all joint combinations between patients and controls analysed by 95% confidence intervals and SPM.

## References

[B1] Adler R. J. Taylor J. E. Worsley K. J. (2007). Applications of Random fields and Geometry: Foundations and Case Studies. Netherlands: Springer.

[B2] Affolter C. Kedzierska J. Vielma T. Weisse B. Aiyangar A. (2020). Estimating Lumbar Passive Stiffness Behaviour from Subject-specific Finite Element Models and *In Vivo* 6DOF Kinematics. J. Biomech. 102, 109681. 10.1016/j.jbiomech.2020.109681 32151379

[B3] Ahmadi A. Maroufi N. Behtash H. Zekavat H. Parnianpour M. (2009). Kinematic Analysis of Dynamic Lumbar Motion in Patients with Lumbar Segmental Instability Using Digital Videofluoroscopy. Eur. Spine J. 18, 1677–1685. 10.1007/s00586-009-1147-x 19727854 PMC2899390

[B4] Aiyangar A. Zheng L. Anderst W. Zhang X. (2015). Apportionment of Lumbar L2-S1 Rotation across Individual Motion Segments during a Dynamic Lifting Task. J. Biomech. 48 (13), 3709–3715. 10.1016/j.jbiomech.2015.08.022 26362687

[B5] Breen A. Breen A. (2016). Accuracy and Repeatability of Quantitative Fluoroscopy for the Measurement of Sagittal Plane Translation and Finite centre of Rotation in the Lumbar Spine. Med. Eng. Phys. 38, 607–614. 10.1016/j.medengphy.2016.03.009 27129784

[B6] Breen A. Breen A. (2020). Dynamic Interactions between Lumbar Intervertebral Motion Segments during Forward Bending and Return. J. Biomech. 102, 109603. 10.1016/j.jbiomech.2020.109603 31964520

[B7] Breen A. Breen A. (2018). Uneven Intervertebral Motion Sharing Is Related to Disc Degeneration and Is Greater in Patients with Chronic, Non-specific Low Back Pain: an *In Vivo*, Cross-Sectional Cohort Comparison of Intervertebral Dynamics Using Quantitative Fluoroscopy. Eur. Spine J. 27 (1), 145–153. 10.1007/s00586-017-5155-y 28555313

[B8] Breen A. Claerbout E. Hemming R. Ayer R. Breen A. (2019a). Comparison of Intra Subject Repeatability of Quantitative Fluoroscopy and Static Radiography in the Measurement of Lumbar Intervertebral Flexion Translation. Sci. Rep. 9, 19253. 10.1038/s41598-019-55905-1 31848427 PMC6917745

[B9] Breen A. C. Muggleton J. M. Mellor F. E. (2006). An Objective Spinal Motion Imaging Assessment (OSMIA): Reliability, Accuracy and Exposure Data. BMC Musculoskelet. Disord. 7 (1), 1–10. 10.1186/1471-2474-7-1 16393336 PMC1351178

[B10] Breen A. C. Teyhen D. S. Mellor F. E. Breen A. C. Wong K. W. N. Deitz A. (2012). Measurement of Intervertebral Motion Using Quantitative Fluoroscopy: Report of an International Forum and Proposal for Use in the Assessment of Degenerative Disc Disease in the Lumbar Spine. Adv. Orthop. 2012, 1–10. 10.1155/2012/802350 PMC3362008 22666606

[B11] Breen A. Hemming R. Mellor F. Breen A. (2019b). Intrasubject Repeatability of *In Vivo* Intervertebral Motion Parameters Using Quantitative Fluoroscopy. Eur. Spine J. 28 (2), 450–460. 10.1007/s00586-018-5849-9 30535658

[B12] Breen A. Mellor F. E. Breen A. C. (2018). Aberrant Intervertebral Motion in Patients with Treatment-Resistant Nonspecific Low Back Pain: a Retrospective Cohort Study and Control Comparison. Eur. Spine J. 27, 2831. 10.1007/s00586-018-5666-1 29926209

[B13] Byrne R. M. Aiyangar A. K. Zhang X. (2020). Sensitivity of Musculoskeletal Model-Based Lumbar Spinal Loading Estimates to Type of Kinematic Input and Passive Stiffness Properties. J. Biomech. 102, 109659. 10.1016/j.jbiomech.2020.109659 32070482

[B14] CLSI (2008). Defining, Establishing and Verifying Reference Intervals in the Clinical Laboratory; Approved Guideline. 3rd Edn. Wayne, PA: Clinical Laboratory Standards Institute.

[B15] Dreischarf M. Shirazi-Adl A. Arjmand N. Rohlmann A. Schmidt H. (2016). Estimation of Loads on Human Lumbar Spine: A Review of *In Vivo* and Computational Model Studies. J. Biomech. 49, 833–845. 10.1016/j.jbiomech.2015.12.038 26873281

[B16] du Rose A. Breen A. (2016). Relationships between Lumbar Inter-vertebral Motion and Lordosis in Healthy Adult Males: a Cross Sectional Cohort Study. BMC Musculoskelet. Disord. 17 (121), 121. 10.1186/s12891-016-0975-1 26964535 PMC4785734

[B17] du Rose A. Breen A. Breen A. (2018). Relationships between Muscle Electrical Activity and the Control of Inter-vertebral Motion during a Forward Bending Task. J. Electromyogr. Kinesiol. 43, 48–54. 10.1016/j.jelekin.2018.08.004 30237131

[B18] El Ouaaid Z. Shirazi-Adl A. Plamondon A. Larivière C. (2013). Trunk Strength, Muscle Activity and Spinal Loads in Maximum Isometric Flexion and Extension Exertions: A Combined In Vivo-computational Study. J. Biomech. 46, 2228–2235. 10.1016/j.jbiomech.2013.06.018 23871523

[B19] Eskandari A. H. Arjmand N. Shirazi-AdlFarahmand A. F. Farahmand F. (2017). Subject-specific 2D/3D Image Registration and Kinematics-Driven Musculoskeletal Model of the Spine. J. Biomech. 57, 18–26. 10.1016/j.jbiomech.2017.03.011 28365064

[B20] Friston K. J. Ashburner J. T. Kiebel S. J. Nichols T. E. Penny W. D. (2007). Statistical Parametric Mapping: The Analysis of Functional Brain Images. London: Elsevier.

[B21] Hajihosseinali M. Arjmand N. Shirazi-Adl A. (2015). Effect of Body Weight on Spinal Loads in Various Activities: A Personalized Biomechanical Modeling Approach. J. Biomech. 48, 276–282. 10.1016/j.jbiomech.2014.11.033 25498363

[B22] Harada M. Abumi K. Ito M. Kaneda K. (2000). Cineradiographic Motion Analysis of normal Lumbar Spine during Forward and Backward Flexion. Spine 25, 1932–1937. 10.1097/00007632-200008010-00011 10908936

[B23] Jones A. C. Wilcox R. K. (2008). Finite Element Analysis of the Spine: Towards a Framework of Verification, Validation and Sensitivity Analysis. Med. Eng. Phys. 30 (10), 1287–1304. 10.1016/j.medengphy.2008.09.006 18986824

[B24] Lee S.-w. Wong K. W. N. Chan M.-k. Yeung H.-m. Chiu J. L. F. Leong J. C. Y. (2002). Development and Validation of a New Technique for Assessing Lumbar Spine Motion. Spine 27 (8), E215–E220. 10.1097/00007632-200204150-00022 11935121

[B25] Linnet K. (1987). Two-stage Transformation Systems for Normalization of Reference Distributions Evaluated. Clin. Chem. 33 (3), 381–386. 10.1093/clinchem/33.3.381 3815802

[B26] Magee J. (2015). Three Dimensional Digital Modelling of Human Spine Anthropometrics and Kinematics from Meta-Analysis. How Relevant Is Existing Anatomical Research? J. Spine 4 (1), 251–257. 10.4172/2165-7939.1000205

[B27] Mellor F. E. Thomas P. Breen A. (2014). Moving Back: The Radiation Dose Received from Lumbar Spine Quantitative Fluoroscopy Compared to Lumbar Spine Radiographs with Suggestions for Dose Reduction. Radiography 20, 251–257. 10.1016/j.radi.2014.03.010 26512196 PMC4579040

[B28] Miyasaka K. Ohmori K. Suzuki K. Inoue H. (2000). Radiographic Analysis of Lumbar Motion in Relation to Lumbosacral Stability. Spine 25 (6), 732–737. 10.1097/00007632-200003150-00014 10752107

[B29] Nagel T. M. Zitnay J. L. Barocas V. H. Nuckley D. J. (2014). Quantification of Continuous *In Vivo* Flexion-Extension Kinematics and Intervertebral Strains. Eur. Spine J. 23, 754–761. 10.1007/s00586-014-3195-0 24487626 PMC3960416

[B30] Okawa A. Shinomiya K. Komori H. Muneta T. Arai Y. Nakai O. (1998). Dynamic Motion Study of the Whole Lumbar Spine by Videofluoroscopy. Spine 23 (16), 1743–1749. 10.1097/00007632-199808150-00007 9728375

[B31] Oxland T. R. (2016). Fundamental Biomechanics of the Spine-What We Have Learned in the Past 25 Years and Future Directions. J. Biomech. 49 (6), 817–832. 10.1016/j.jbiomech.2015.10.035 26706717

[B32] Panjabi M. M. (1992). The Stabilizing System of the Spine. Part I. Function, Dysfunction, Adaptation, and Enhancement. J. Spinal Disord. 5 (4), 383–389. 10.1097/00002517-199212000-00001 1490034

[B33] Papi E. Bull A. M. J. McGregor A. H. (2020). Alteration of Movement Patterns in Low Back Pain Assessed by Statistical Parametric Mapping. J. Biomech. 100, 109597. 10.1016/j.jbiomech.2019.109597 31928738 PMC7001037

[B34] Papi E. Bull A. M. J. McGregor A. H. (2018). Is There Evidence to Use Kinematic/kinetic Measures Clinically in Low Back Pain Patients? A Systematic Review. Clin. Biomech. 55, 53–64. 10.1016/j.clinbiomech.2018.04.006 PMC6161016 29684790

[B35] Pataky T. C. (2016). rft1d: Smooth One-Dimensional Random Field Upcrossing Probabilities inPython. J. Stat. Soft. 71, i07. 10.18637/jss.v071.i07

[B36] Pataky T. Vanrenterghem J. Robinson M. A. (2016). The Probability of False Positives in Zero-Dimensional Analyses of One-Dimensional Kinematic, Force and EMG Trajectories. J. Biomech. 49, 1468. 10.1016/j.jbiomech.2016.03.032 27067363

[B37] Pavlova A. V. Meakin J. R. Cooper K. Barr R. J. Aspden R. M. (2018). Variation in Lifting Kinematics Related to Individual Intrinsic Lumbar Curvature: an Investigation in Healthy Adults. BMJ Open Sport Exerc. Med. 4, e000374. 10.1136/bmjsem-2018-000374 PMC6059291 30057776

[B38] Retailleau M. Colloud F. (2020). New Insights into Lumbar Flexion Tests Based on Inverse and Direct Kinematic Musculoskeletal Modeling. J. Biomech. 105, 109782. 10.1016/j.jbiomech.2020.109782 32423539

[B39] Takayanagi K. Takahashi K. Yamagata M. Moriya H. Kitahara H. Tamaki T. (2001). Using Cineradiography for Continuous Dynamic-Motion Analysis of the Lumbar Spine. Spine 26 (17), 1858–1865. 10.1097/00007632-200109010-00008 11568694

[B40] Teyhen D. S. Flynn T. W. Childs J. D. Kuklo T. R. Rosner M. K. Polly D. W. (2007). Fluoroscopic Video to Identify Aberrant Lumbar Motion. Spine 32 (7), E220–E229. 10.1097/01.brs.0000259206.38946.cb 17414897

[B41] To D. Breen A. Breen A. Mior S. Howarth S. J. (2020). Investigator Analytic Repeatability of Two New Intervertebral Motion Biomarkers for Chronic, Nonspecific Low Back Pain in a Cohort of Healthy Controls. Chiropr Man. Therap 28 (62), 1–9. 10.1186/s12998-020-00350-5 PMC7685540 33228737

[B42] Van Dillen L. R. Sahrmann S. A. Norton B. J. (2013). “The Kinesiopathological Model and Mechanical Low Back Pain,” in Spinal Control: The Rehabilitation of Low Back Pain. Editors Hodges P.W. Cholewicki J. Van Dieen J. (Edinburgh: Churchill Livingstone Elsevier), 89–98. 10.1016/b978-0-7020-4356-7.00008-2

[B43] Wallwork T. L. Stanton W. R. Freke M. Hides J. A. (2009). The Effect of Chronic Low Back Pain on Size and Contraction of the Lumbar Multifidus Muscle. Man. Ther. 14 (5), 496–500. 10.1016/j.math.2008.09.006 19027343

[B44] Widmer J. Fornaciari P. Senteler M. Roth T. Snedeker J. G. Farshad M. (2019). Kinematics of the Spine under Healthy and Degenerative Conditions: a Systematic Review. Ann. Biomed. Eng. 47, 1491–1522. 10.1007/s10439-019-02252-x 30937563

[B45] Wong K. W. N. Luk K. D. K. Leong J. C. Y. Wong S. F. Wong K. K. Y. (2006). Continuous Dynamic Spinal Motion Analysis. Spine 31 (4), 414–419. 10.1097/01.brs.0000199955.87517.82 16481951

[B46] Zander T. Rohlmann A. Klöckner C. Bergmann G. (2002). Comparison of the Mechanical Behavior of the Lumbar Spine Following Mono- and Bisegmental Stabilization. Clin. Biomech. 17, 439–445. 10.1016/s0268-0033(02)00040-2 12135545

[B47] Zanjani-Pour S. Meakin J. R. Breen A. Breen A. (2018). Estimation of *In Vivo* Inter-vertebral Loading during Motion Using Fluoroscopic and Magnetic Resonance Image Informed Finite Element Models. J. Biomech. 70, 134–139. 10.1016/j.jbiomech.2017.09.025 29037442

