# Supplementary figures and images for: A Reference Database of Standardised Continuous Lumbar Intervertebral Motion Analysis for Conducting Patient-Specific Comparisons
Source: Front Bioeng Biotechnol. 2021 Sep 27;9:745837. doi: 10.3389/fbioe.2021.745837 (PMC8503612; doi:10.3389/fbioe.2021.745837)

## Slide 1
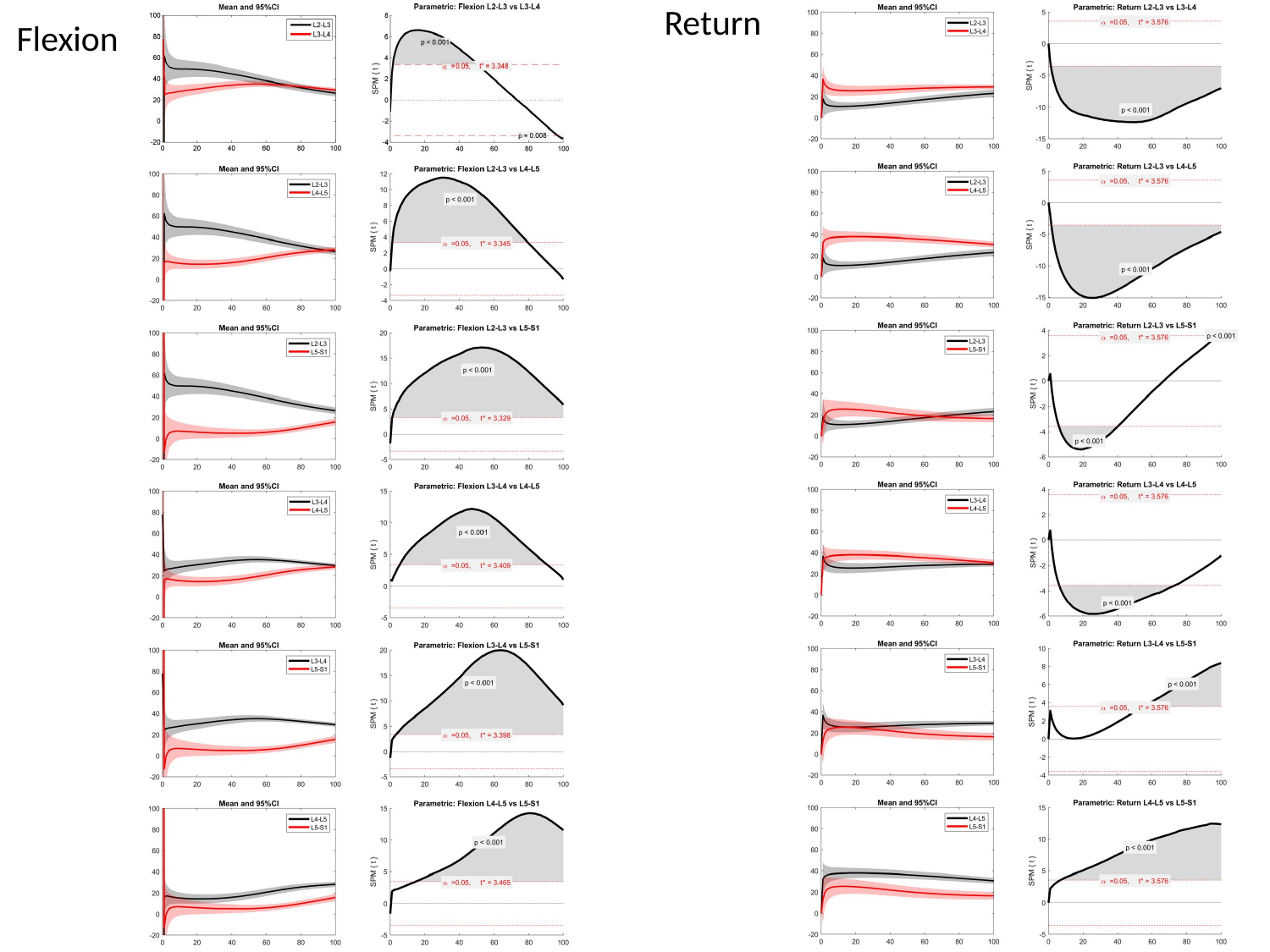

Return
Flexion

Supplement: Supplementary file 1 [file Presentation1.PPTX]

## Slide 1
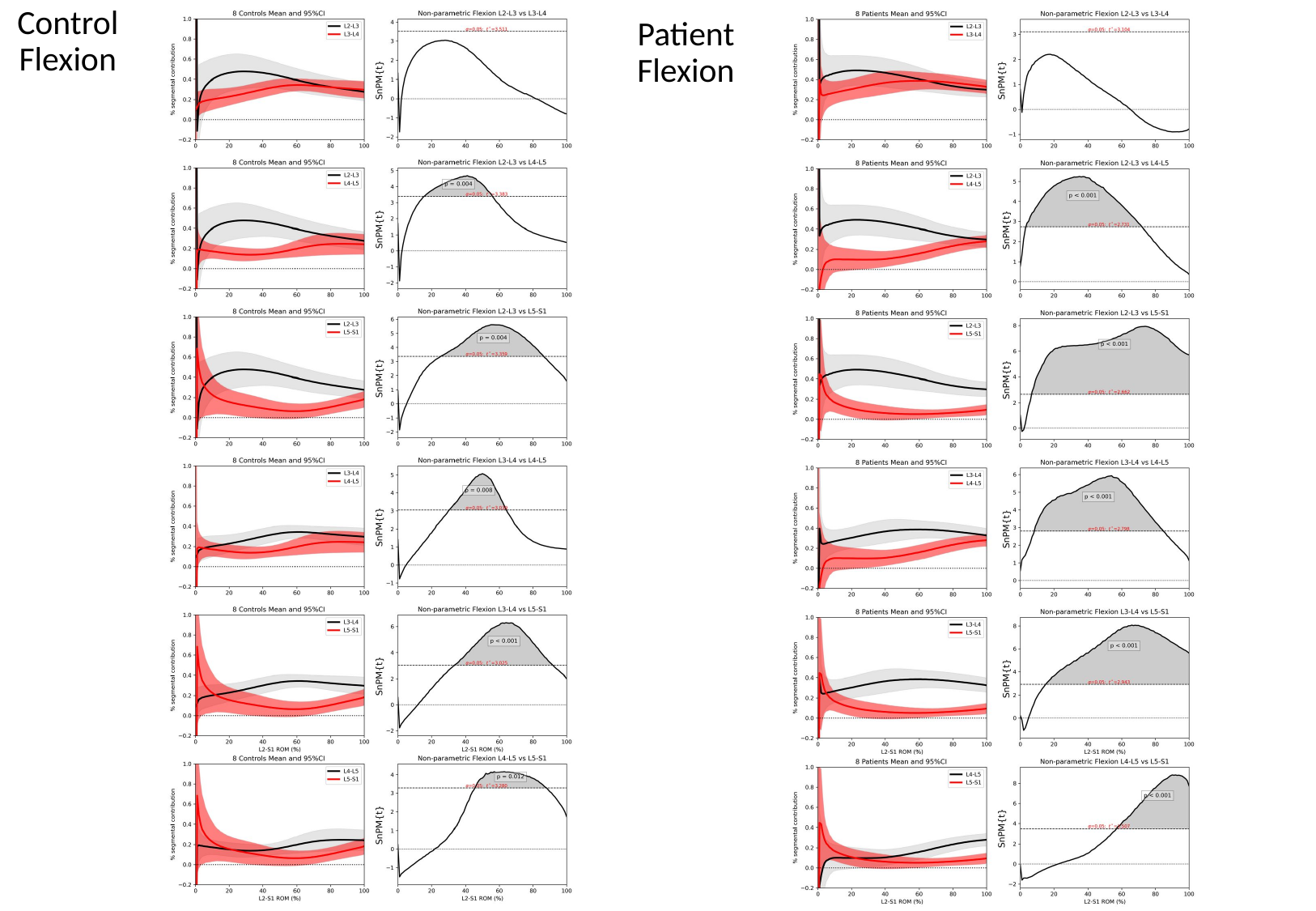

Control Flexion
Patient Flexion

## Slide 2
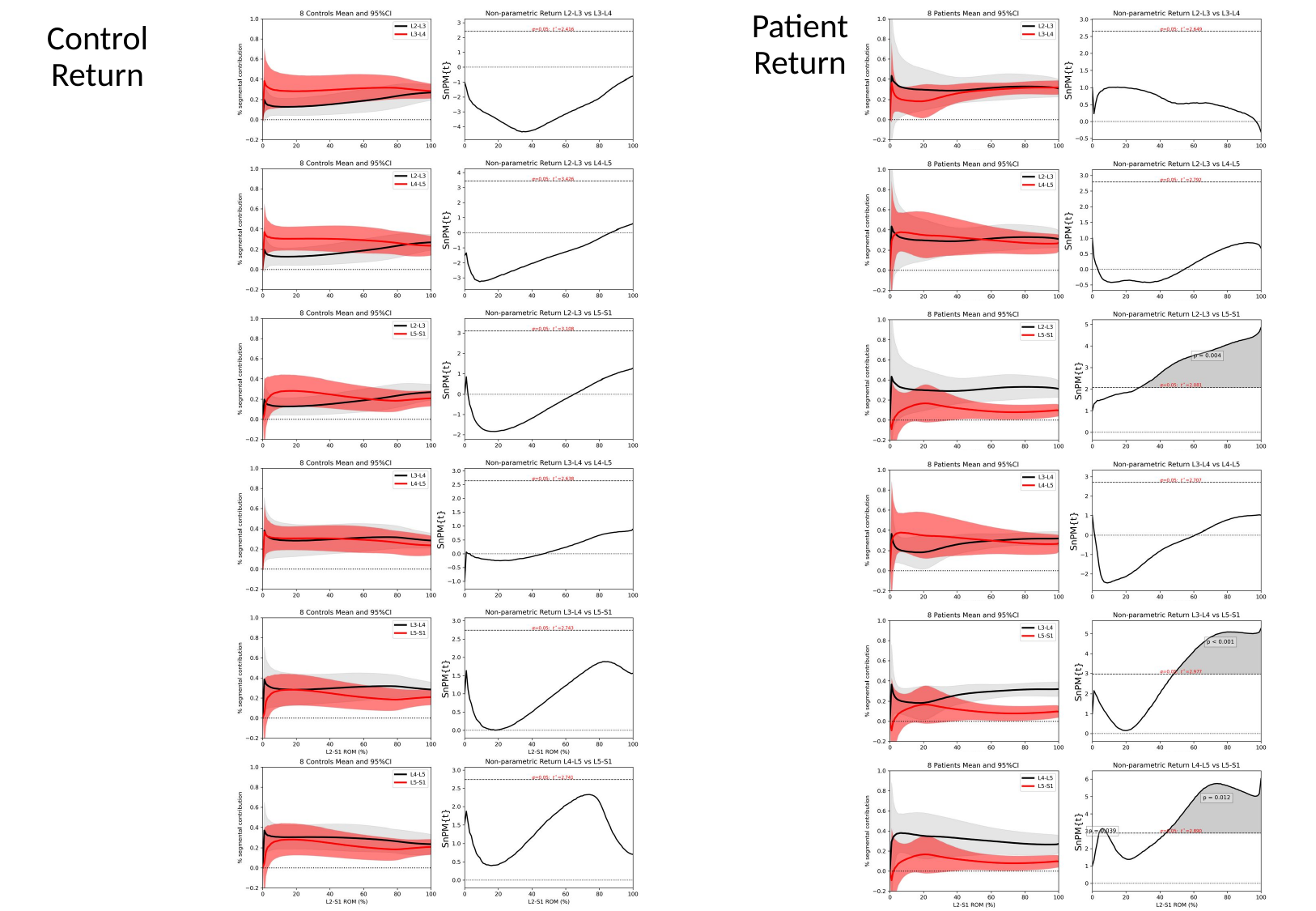

Patient Return
Control Return

Supplement: Supplementary file 3 [file Presentation2.PPTX]
